# Spatiotemporal immune atlas of the first clinical-grade, gene-edited pig-to-human kidney xenotransplant

**DOI:** 10.21203/rs.3.rs-2382345/v1

**Published:** 2023-01-09

**Authors:** Matthew D. Cheung, Rebecca Asiimwe, Elise N. Erman, Christopher F. Fucile, Shanrun Liu, Chiao-Wang Sun, Vidya Sagar Hanumanthu, Harish C. Pal, Emma D. Wright, Gelare Ghajar-Rahimi, Daniel Epstein, Babak J. Orandi, Vineeta Kumar, Douglas J. Anderson, Morgan E. Greene, Markayla Bell, Stefani Yates, Kyle H. Moore, Jennifer LaFontaine, John T. Killian, Gavin Baker, Jackson Perry, Rhiannon Reed, Shawn C. Little, Alexander F. Rosenberg, James F. George, Jayme E. Locke, Paige M. Porrett

**Affiliations:** 1 Department of Surgery, University of Alabama at Birmingham; Birmingham, AL, USA; 2 Informatics Institute, University of Alabama at Birmingham; Birmingham, AL, USA; 3 Department of Biochemistry and Molecular Genetics, University of Alabama at Birmingham; Birmingham, AL, USA; 4 Flow Cytometry & Single Cell Core Facility, University of Alabama at Birmingham; Birmingham, AL, USA; 5 Department of Medicine, University of Alabama at Birmingham; Birmingham, AL, USA; 6 Department of Microbiology, University of Alabama at Birmingham; Birmingham, AL, USA

## Abstract

Pig-to-human xenotransplantation is rapidly approaching the clinical arena; however, it is unclear which immunomodulatory regimens will effectively control human immune responses to pig xenografts. We transplanted a gene-edited pig kidney into a brain-dead human recipient on pharmacologic immunosuppression and studied the human immune response to the xenograft using spatial transcriptomics and single-cell RNA sequencing. Human immune cells were uncommon in the porcine kidney cortex early after xenotransplantation and consisted of primarily myeloid cells. Both the porcine resident macrophages and human infiltrating macrophages expressed genes consistent with an alternatively activated, anti-inflammatory phenotype. No significant infiltration of human B or T cells into the porcine kidney xenograft was detected. Altogether, these findings provide proof of concept that conventional pharmacologic immunosuppression is sufficient to restrict infiltration of human immune cells into the xenograft early after compatible pig-to-human kidney xenotransplantation.

Kidney allotransplantation is a life-saving therapy for people with end-stage kidney disease, but there are not enough human kidneys to meet the growing demand^1,2^. Xenotransplantation is a promising solution to increase organ supply; however, it is unclear whether immunomodulatory strategies currently used in allotransplantation will effectively control human immune responses to xenografts. Although studies of porcine kidney xenografts in nonhuman primate (NHP) recipients suggest that many mechanisms of immune injury are conserved between allotransplant and xenotransplant recipients^3^, crossmatch-compatible xenotransplantation has not yet been achieved in NHPs despite genetic knockdown of carbohydrate antigens that promote crossmatch compatibility between pig cells and human sera^4–6^. While short- to intermediate-term graft survival is possible in limited numbers of incompatible xenotransplant NHP recipients, overall recipient survival is poor due to complications of intensive immunosuppression^7^. Crossmatch-compatible transplantation remains thus a primary goal of current xenotransplantation efforts^8^, as this strategy will likely result in safest outcomes for human transplant recipients. However, immunomodulatory strategies which provide effective immune control in compatible pig-to-human xenotransplantation cannot be assessed in incompatible NHP models. Evaluation of immune control in human recipients of compatible porcine xenografts is therefore critically needed in order to ensure recipient safety in forthcoming first-in-human clinical trials of xenotransplantation.

We developed a preclinical model of pig-to-human kidney xenotransplantation to test the hypothesis that current standard-of-care pharmacologic immunosuppression would control human immune responses in the porcine kidney early after crossmatch-compatible xenotransplantation. As previously reported, porcine kidney xenografts were procured from a domestic pig with 10 genetic modifications^9^ and transplanted into a nephrectomized, crossmatch-compatible, brain-dead human recipient (Extended Data Fig. 1a)^10^. Immunomodulation of the recipient was established through both genetic modification of the porcine kidney and conventional pharmacologic immunosuppression. In addition to knockdown of the growth hormone receptor and three carbohydrate xenoantigens (*GGTA1, B4GALNT2, CMAH*), the porcine kidneys expressed human transgenes (*CD55, CD46, THBD, PROCR, CD47, HMOX1*) intended to reduce inflammation and prevent thrombotic complications within the kidney. Pharmacologic immunosuppression consisted of induction therapy with methylprednisolone, anti-thymocyte globulin, and rituximab, while maintenance immunosuppression included tacrolimus, mycophenolate mofetil, and prednisone^10^. The kidney xenografts made urine but did not clear creatinine^10^; H&E sections of the xenografts revealed no evidence of acute cellular rejection or binding of IgM, IgG, or complement proteins^10^. The experiment was terminated approximately 74 hours after transplant due to hemodynamic instability^10^.

Sequential needle core biopsies of the gene-edited porcine kidneys were taken immediately prior to transplantation, *in situ* on postoperative days 1 and 3, and immediately prior to explant (Extended Data Fig. 1b). Biopsies were analyzed using spatial transcriptomics and single-nuclear RNA-sequencing (snRNA-seq) approaches. As detection of immune populations can be limited in samples analyzed by snRNA-seq^11^, we also performed single-cell RNA-seq (scRNA-seq) on CD45+ immune cells that were FACS-enriched from the explanted xenografts (Extended Data Fig. 1b). In order to distinguish human from porcine cells, we aligned all sequenced reads to a custom human-porcine hybrid reference genome and annotated clusters based on established marker genes^12^.

As discrimination of porcine from human immune cells was necessary for this study, we assessed the specificity of our mapping with three different approaches. First, we aligned independent control biopsies from human and pig kidneys to the hybrid reference genome and found that key immune genes mapped to the appropriate species reference (Extended Data Fig. 2). Although these analyses evaluated the performance of our pipeline on samples derived from a single species, we hypothesized that pipeline performance might differ for mixed species samples (i.e. the xenograft). We therefore evaluated the mapping specificity of individual reads sequenced from CD45+ immune cells sorted from the explanted xenograft. Cells were initially clustered according to both cell type and species as individual genes had species-specific annotation after alignment to the hybrid reference (Extended Data Fig. 3). We found that 97.8% of 18,833,529 transcripts recovered from 6,513 immune cells associated with a single species, and 98.8% of cells possessed >90% of transcripts from a single species (Extended Data Fig. 4). These analyses further revealed that ~10% of reads in pig macrophages mapped to 17 human genes (0.27% of human genes), likely as a consequence of high sequence homology (Extended Data Fig. 5). Finally, to assess the impact of homology on the species assignment of individual cells, we employed an alternative mapping strategy using custom modified reference genomes composed of more species-specific genes (see Methods & Extended Data Fig. 6). We found a high correlation in the species assignment of cellular barcodes between the two methods (Extended Data Fig. 7), suggesting that the presence of gene homology did not significantly impact the species assignment for most cell types when using the hybrid reference genome. Collectively, these analyses highlighted the benefit of using the entire transcriptome to make cell assignments in lieu of individual genes that may not be specific to species or cell type.

We therefore used Cell2location^13^ to deconvolute our spatial transcriptomic data from the xenograft biopsies into pig and human cell types based on input of reference transcriptomes. Reference transcriptomes were derived from sorted CD45+ cells and parenchymal cells from the porcine xenograft biopsies (Extended Data Fig. 8). We restricted the input to Cell2location to reference transcriptomes recovered from the xenografts given the unknown impact of immunosuppression and/or ischemic injury on immune transcriptomes. While porcine immune cells were readily detected in pre- and post-transplant xenograft biopsies, human immune cell types were not appreciably detected until post-transplant day 3 and were far less abundant than porcine immune cells (Fig. 1). As would be expected in the setting of effective T- and B-cell depletion^10^, all human immune cells detected in the xenograft biopsies derived from myeloid lineages (Fig. 1 & Extended Fig. 9). Because human adaptive immune cells were not well represented among CD45+ immune cells sorted from the xenograft (Extended Figs. 3 & 8), we attempted to identify human T and B cells by expression of multiple individual T- and B-cell genes known to be relatively specific to these cell types (Fig. 1 & Extended Fig. 9). Altogether, these experiments reveal limited infiltration of the renal cortex by human immune cells.

As macrophages represented the most prevalent immune cell type in our biopsy data and are known to impact allotransplant outcomes^14,15^, we interrogated macrophage activation state by examining expression of classically activated M1 and alternatively activated M2 genes in pig and human macrophage subsets among CD45+ cells sorted from the explant (Extended Data Table 1)^16^. We found increased individual gene expression (Fig. 2a,b) and composite gene expression scores of M2 compared to M1 genes in both pig and human macrophages (Fig. 2c). Although differences in gene expression between the species precluded comprehensive formal comparison of donor- versus recipient-derived macrophages, we performed a limited comparison of pro- and anti-inflammatory cytokines and select genes of interest known to be important in macrophage activation and function^17^ (Fig. 2d). Collectively, these data suggest that macrophages populating the kidney xenograft express a more alternatively activated, anti-inflammatory transcriptome, independent of species.

Altogether, this study of the immune census of porcine kidney xenografts after pig-to-human xenotransplantation suggests that current genetic and pharmacologic immunomodulation strategies can prevent early T and B cell infiltration of the xenograft and modulate inflammation provoked by innate immune responses. Despite these encouraging results, our study has several limitations. First, we were not able to determine the impact of immunomodulatory strategies at later time points due to the short duration of the experiment. Second, our data do not distinguish the specific impact of pharmacologic interventions from genetic edits within the pig kidney. Third, we may have overestimated infiltrating human cell number as we could neither distinguish cells in the kidney interstitium from potential blood contaminants nor correct for alignment error for homologous genes. We attempted to overcome these limitations by using the whole transcriptome to make species and cell type assignments to reduce the impact of homologous genes on our cell calls. We also focused our analyses on cells unlikely to represent blood contaminants (i.e. macrophages). Nevertheless, even in the event that we have inaccurately identified 20% of pig macrophages as human cells (Extended Fig. 7), these data still suggest that infiltration of the xenograft by human immune cells known to promote rejection (i.e. T cells) or chronic injury (i.e. inflammatory macrophages) is limited. Finally, although we observed diminished pro-inflammatory gene signatures in infiltrating macrophages, the clinical benefit of attenuation of inflammation in these macrophages is unclear, and additional studies in living human recipients where longer-term data can be acquired will be needed. Despite these limitations, our study provides proof of concept that the human adaptive and innate immune responses to a porcine xenograft can be controlled by currently available genetic and/or conventional pharmacologic interventions. Our data thus address a critical knowledge gap about the safety and feasibility of porcine kidney xenotransplantation and inform immunosuppression design for upcoming clinical trials.

## Methods:

### Porcine kidney xenotransplantation

Pig-to-human kidney xenotransplantation in the preclinical human decedent model (“Parsons Model”) was conducted in accordance with IRB-300004648 as previously described^10^. In brief, kidney procurement from a pig with 10 gene edits (“10-GE”) was performed using aseptic technique in a surgical suite adjacent to a facility free of designated pathogens on the Xenotransplantation Procurement Campus (XPC) of the University of Alabama at Birmingham Heersink School of Medicine (UAB)^10^. Oversight of all activities at the UAB XPC was provided by the Institutional Animal Care and Use Committee (IACUC-22015).

The recipient of the porcine kidney xenograft was a 57-year-old brain-dead human male ruled out for organ donation, whose next-of-kin authorized research and transport to the Legacy of Hope donor recovery center at the University of Alabama at Birmingham^10^. After completion of a negative prospective crossmatch with the donor pig, the recipient underwent native nephrectomies followed by transplantation of right and left 10-GE porcine kidney xenografts^10^. The brain-dead human recipient was maintained in an operating room in the donor recovery center and supported by various intensive care interventions (e.g., ventilation, pharmacologic pressors, etc.) until termination 74 hours after transplantation of the porcine kidney xenografts^10^. Pharmacologic immunosuppression consisted of induction therapy with methylprednisolone, anti-thymocyte globulin (ATG), and rituximab (anti-CD20), while maintenance immunosuppression included tacrolimus, mycophenolate mofetil and prednisone (see table below and reference 10). Additional methylprednisolone doses were given for management of brain death.

**Table:** 

Immunosuppressive Medication	POD 0	POD 1	POD 2	POD 3

Anti-Thymocyte Globulin (Rabbit)	175 mg	175 mg	175 mg	--

Rituximab	1800 mg	--	--	--

Tacrolimus	--	1 mg AM	1 mg AM	2 mg AM
	1 mg PM	1 mg PM	2 mg PM	--

Mycophenolate Mofetil	--	1000 mg AM	1000 mg AM	1000 mg AM
	2000 mg PM	1000 mg PM	1000 mg PM	--

Methylprednisolone	500 mg	250 mg	125 mg	90 mg

### Sample collection

#### 1) Porcine kidney xenograft biopsies

Biopsies of the 10-GE porcine kidney were collected prior to transplantation and at 24, 72, and 74 hours post-transplantation using a core biopsy needle. All post-transplantation biopsies were collected from the xenograft in situ in the brain-dead human recipient. Samples were placed into PBS on ice and transferred to the laboratory where they were immediately embedded in Optimal Cutting Temperature and flash frozen in a 2-methylbutane container surrounded by liquid nitrogen. Frozen samples were stored in blocks at −80°C until sectioned. A section of each biopsy was placed on a Visium gene expression slide (10X Genomics) for spatial transcriptomic analysis. The remainder of each biopsy was committed to nuclear isolation and single-nuclear RNA-seq as below.

#### 2) Porcine kidney xenograft explant processing and immune cell isolation

A section of the explanted pig kidney was chosen to represent the kidney from the cortex through the papillary region. The kidney section was finely minced using a razor blade and placed into a medium containing 16.7 units per mL Liberase^™^ (Millipore Sigma, Roche; Indianapolis) in RPMI 1640 (Gibco^™^ ThermoFisher Scientific; Grand Island, NY) for 30 minutes at 37°C. The reaction was stopped by adding phosphate-buffered saline (PBS) (Gibco^™^ ThermoFisher Scientific; Bleiswijk, The Netherlands) with 1% w/v bovine serum albumin (BSA) (Fisher Bioreagents^™^ of Fisher Scientific; Fair Lawn, NJ) and then tissue was pulled through an 18-gauge syringe to dissociate the remaining tissue to a single-cell suspension. Cells were pelleted at 500g for 5 minutes and resuspended in ACK lysis buffer (ThermoFisher Scientific) for 2 minutes to lyse red blood cells. Cells were washed with 45 mL of PBS and then stained with 1 μL/1×10 cells^6^ Aqua fixable viability dye (Invitrogen Life Scientific^™^ of ThermoFisher Scientific; Eugene, OR). Cells were incubated in 5 μL/100 μL cell suspension Human TruStain FcX, Fc Receptor blocking solution, (Biolegend^®^ Inc.; San Diego, CA) for 10 minutes at room temperature and then stained with 0.5 mg/mL anti-human CD45 FITC (Biolegend^®^ Inc.; San Diego, CA) and 10 μL/100 μL cell suspension of mouse anti-pig CD45-Alexa Fluor^®^ 647 conjugate antibody (Bio-Rad Laboratories, Inc). Cells were sorted using a BD FACSAria for human and pig CD45+ cells into a tube containing PBS (Gibco^™^ ThermoFisher Scientific; Bleiswijk, The Netherlands) + 0.04% w/v BSA.

#### 3) Peripheral Blood Mononuclear Cells (porcine & human)

Blood was collected in EDTA-K2 tubes vacutainers (BD and Company; Franklin, NJ). PBMC isolation was performed at room temperature until red blood cell (RBC) lysis. Equal volumes of DPBS without Ca2+ or Mg2+ (Gibco^™^ ThermoFisher Scientific; Grand Island, NY) and 2% FBS (Gemini Bio; West Sacramento, CA) was added to the whole blood and mixed (i.e. 5 mL of whole blood was diluted with 5 mL of buffer). The manufacturer’s instructions were followed in order to isolate PBMCs using LymphoprepTM (STEMCELL Technologies; Vancouver, BC) and either a Sepmate^™^-15 (StemCell Technologies; Vancouver, BC) or a Sepmate^™^-50 (StemCell Technologies; Vancouver, BC), depending on the blood volume. Once the PBMCs were isolated, the cells were washed with buffer two times, once at 300xrcf for 8 minutes and once at 120xrcf for 10 minutes with no brake. After removing the wash buffer, RBCs were lysed using 4 mL of room temperature ACK lysis buffer (Quality Biological; Gaithersburg, MD) for 2 minutes on ice and then the ice cold DPBS was added to the 14 mL mark and mixed. Cells were pelleted by centrifugation, 400xrcf for 5 minutes at 4°C. The buffer was removed and 0.5–1 mL fresh, ice cold DPBS without Ca2+ and Mg2+ was added and the cells were suspended. Cells were counted and delivered to the UAB Single Cell core, and scRNA-seq was performed.

#### 4) Human kidney

The native kidneys which were removed from the brain-dead recipient prior to xenotransplantation were offered for allotransplantation but ultimately declined. After exhaustion of the transplant list, the kidneys were transported to the laboratory and processed for spatial transcriptomics analysis as described below.

#### 5) Control pig kidneys

Porcine control kidneys were recovered from 1) a 208-day-old Chester White Cross wild-type sow (Identification number 817D), weighing 154 kgs, and 2) an 8-day-old Chester White Cross 10-GE male piglet (identification number 817D-1) weighing 1.3 kg at the UAB XPC. The kidneys were transported to the laboratory and processed for spatial transcriptomics analysis as described below.

### Spatial Transcriptomics – sample preparation

Frozen biopsy OCT blocks were equilibrated to −10°C before use. Using a cryostat, a 10 μm section was placed onto a Visium Spatial Gene Expression Slide (10X Genomics) and processed according to the manufacturer’s protocol. In summary, slides were fixed in methanol for 30 minutes and stained with Hematoxylin and Eosin. Brightfield images were taken using a Keyence BZ-X700 microscope. Slides were placed in specialized slide cassette holders and permeabilization enzyme was added for 12 minutes at 37°C to release the RNA onto the slide. Using the captured RNA, cDNA and subsequently second-strand DNA was created and amplified. Libraries were generated and sent for paired-end sequencing on the NovaSeq6000 (Illumina) for a depth of 50,000 reads per capture spot.

### Nuclear isolation for single-nuclear RNA-sequencing

After a section was taken for spatial transcriptomics, the remaining xenograft biopsy tissue was thawed from OCT and washed with PBS. Nuclei were subsequently isolated using Nuclei Lysis Buffer containing Nuclei Isolation Kit: Nuclei EZ Prep Buffer (Sigma) supplemented with cOmplete ULTRA Tablets (Sigma) and SUPERase IN (Thermo) and Promega RNAsin Plus nuclease inhibitors. Tissue was minced into <1 mm pieces in 2 mL of Nuclei Lysis Buffer. Samples were transferred to a Dounce homogenizer (Kimble) and homogenized. An additional 2 mL of Nuclei Lysis Buffer was added to the sample and incubated for 5 minutes on ice. Samples were passed through a 40 μm filter into a 50 mL conical tube. Samples were centrifuged at 500 × g for 5 minutes at 4°C. The supernatant was removed and the pellet was washed with 4 mL of Nuclei Lysis Buffer containing 1% Bovine Serum Albumin for 5 minutes on ice. Samples were centrifuged at 500 × g for 5 minutes at 4°C. Samples were passed through a 5 μm filter into a 50 mL conical tube and then centrifuged again. Nuclei were resuspended in a solution containing phosphate-buffered saline (PBS), 1% BSA, and 0.1% RNAse inhibitor.

### Preparation of gel emulsion microdroplets and single-cell/single-nuclear RNA sequencing

Suspensions of single cells (or nuclei) were placed on ice and transferred to the UAB Flow Cytometry and Single Cell Core where they were immediately processed using a Chromium 3’ Single-Cell RNA reagent kit v3 (10X Genomics) according to the manufacturer’s protocol. In summary, cells or nuclei were counted and loaded onto the Chromium Controller (10X Genomics), and gel emulsion microdroplets were prepared. cDNA was generated and amplified from mRNA collected within each microdroplet. Libraries were generated and sent for paired-end sequencing on a NovaSeq6000 (Illumina) for a depth of 20,000 reads per cell.

### Generation of the hybrid human-pig genome reference

The human GRCh38 (GCA_000001405.28) and porcine *Sus scrofa* 11.1 (GCA_000003025.6) fasta and gene annotation (gtf) files were downloaded from Ensembl. Gtf files were filtered according to the standard 10X Genomics protocol (https://support.10xgenomics.com/single-cell-gene-expression/software/pipelines/latest/advanced/references), including protein-coding, lincRNA, antisense, and immunoglobulin genes. The two genomes were merged using the Cell Ranger version 6.0 *mkref* function to create the hybrid hg38-ss11 genome.

### Analyses using the hybrid human-pig reference genome:

#### 1) Datasets:

**Figure 1.**
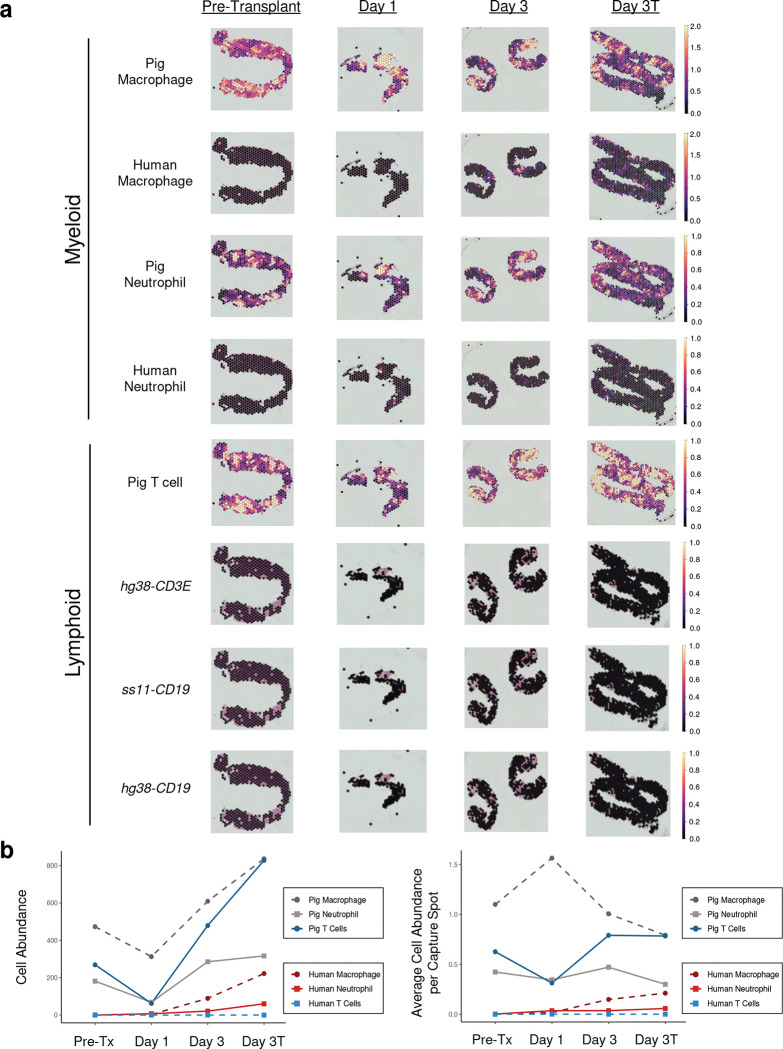
Limited infiltration of the porcine kidney xenograft by human immune cells. Spatial transcriptomics was performed on serial needle core biopsies of 10-GE porcine kidneys before and after transplantation into a brain-dead human recipient. Biopsies were obtained from either the right (pre-transplant and Day 3 samples) or left (Day 1 and Day 3T) xenografts. Cell type signatures were identified from reference transcriptomes using Cell2location or expression of individual marker genes (*CD3E* and *CD19*). **a**) *Top*: Human myeloid cells are detected in biopsies of the porcine kidney xenograft three days after transplantation. Capture spot color corresponds to cell abundance, and color scales to the right of each spatial plot indicate cell abundance. Note that scaling is conserved across time for a given cell type but differs between macrophages (cell abundance range: 0–2) and neutrophils (cell abundance range: 0–1). Visualization of cell abundance in a given capture spot is thus capped at 1 or 2 cells. *Bottom:* No detection of human T or B cells in xenograft biopsies at any time point. **b**) Calculated total (*left*) and normalized (*right*) cell abundance for various immune cell types in the indicated biopsies. For clarity, quantification of B cells is not shown. Note that cell abundance for human T cells and all B cells was imputed from expression of *hg38-CD3E*, *ss11-CD19*, and *hg38-CD19* genes as shown in (**a**). “Pre-tx” = pre-transplant. Day 3T biopsy was taken on post-transplant day 3 at study termination.

**Figure 2.**
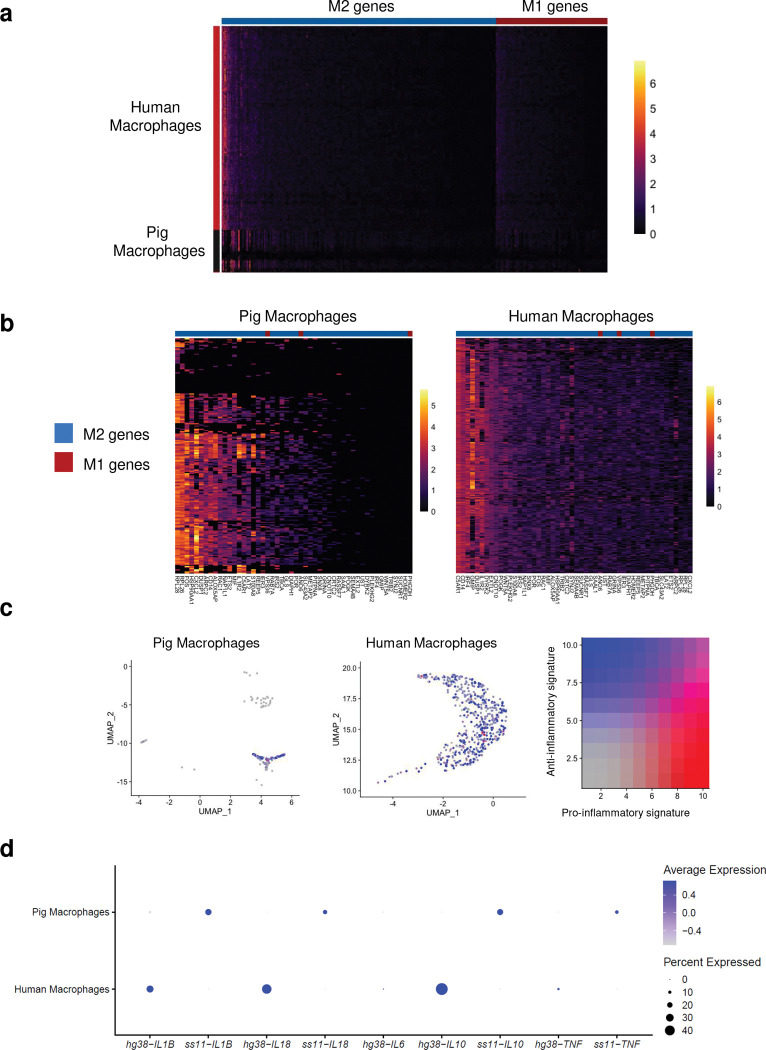
Predominance of M2-like macrophages in the porcine kidney xenograft. scRNA-seq was performed on CD45+ immune cells sorted from the right porcine kidney xenograft at explant (see Extended Fig. 3), and macrophage clusters were selected for analysis. **a**) Expression of M1 (red) and M2 (blue) genes in human and pig macrophages (see Extended Data Table 1 and ref 16 for full gene list). **b**) Expanded view of top 50 most highly expressed M1 and M2 genes in each species. Note M2 > M1 genes for both species. **c**) Composite gene expression score of pig and human macrophages of M1-like pro-inflammatory (red) and M2-like anti-inflammatory (blue) gene signatures (ref 16). UMAPs were generated from re-clustering of macrophage clusters selected from Extended Fig. 3. **d**) Expression of select anti- and pro-inflammatory cytokine genes in pig and human macrophages. Average gene expression is visualized such that the mean of the scaled expression dataset is set at 0 with a standard deviation of 1. *ss11-IL6* was not detected.

- scRNA-seq library prepared from CD45+ immune cells sorted from the explanted xenograft (Fig. 1 [Cell2location reference], Fig. 2, Extended Data Figs. 3–5, 7 & 8);
- snRNA-seq libraries prepared from single nuclei isolated from core biopsies of the porcine kidney xenograft (Fig. 1 [Cell2location reference]; Extended Data Fig. 8);
- Spatially barcoded libraries prepared from core biopsies of the porcine kidney xenograft (Fig. 1; Extended Data Fig. 9).
- Spatially barcoded libraries prepared from control kidney samples, including human kidney, 10-GE pig kidney, and wild-type pig kidney (Extended Data Fig. 2).

#### 2) scRNA-seq data processing and analysis of CD45+ cells sorted from the porcine xenograft at explant:

Cell Ranger (v.6.1.1) was used to pre-process sequenced reads aligned against the hybrid human-pig genome reference to generate raw count matrices. Downstream analysis on the filtered UMI expression profile for each droplet was completed using R (v.4.2.1) and Seurat^18^ (v.4.2.0) using default parameters unless otherwise specified. Before conducting additional analyses, background noise from ambient RNA was removed using SoupX^19^ (v.1.6.1). The overall contamination fraction (rho) was parameterized using the *autoEstCont* function to remove > 2% background contamination in our dataset. The SoupX-corrected count matrix was then loaded into R using the *Read10X* function and was further used to create a Seurat object. Cells with < 200 unique features, > 3000 unique features, and > 12% mitochondrial gene expression were filtered. Features expressed in < 5 cells, and cells with doublet scores > 0.3 were also removed. Data were then normalized by a scale factor of 10,000 and log1p-transformed using the *LogNormalize* function. We then identified the top 3000 variable genes, ranked by coefficient of variation, using *FindVariableFeatures*. Using the variable genes, we scaled and centered the genes across the cells using the *ScaleData* function followed by the identification of principal components (*RunPCA*). Thirty principal components were found and used to construct a k-nearest neighbor (KNN) graph using *FindNeighbors*. Clustering was subsequently performed using *FindClusters* which employs a shared nearest neighbor (SNN) modularity optimization based clustering algorithm. Cluster information was used as input into the uniform manifold approximation and projection algorithm (*RunUMAP*) which further aided the visualization of cell manifolds in a low-dimensional space. We ran Seurat’s implementation of the Wilcoxon rank-sum test (*FindMarkers*) to identify differentially expressed genes in each cluster. The expression of cluster-specific canonical markers was used to annotate each cell cluster.

Module scores were determined for the Seurat object using the *AddModuleScore* function with the M1/pro-inflammatory or M2/anti-inflammatory gene lists as inputs (Extended Data Table 1 & ref 16). A feature plot was generated using *FeaturePlot* with the features set as the calculated module scores from each gene list.

#### 3) snRNA-seq data processing and analysis of parenchymal cells from needle core biopsies of the porcine kidney xenograft

Sequenced reads from nuclei recovered from the four needle core biopsies of the porcine xenograft in situ (see above) were processed using Cell Ranger (v.6.1.1) and aligned to the hybrid human-pig reference genome. Generated snRNA-seq counts from each sample were further processed using Seurat (v.3.2.3) and its associated dependencies. Specifically, filtered UMI counts from each time point were imported into R using the *Read10X* function and structured into sample-specific Seurat objects (*CreateSeuratObject*). Generated objects from each time point were labeled with unique group identifiers and merged into a single object using Seurat’s *merge()* function while including genes detected in at least 5 cells. The merged dataset was further filtered to retain cells with unique feature counts over 200 or under 2500, and cells with a fraction of nuclear-encoded mitochondrial genes <15%. Data were normalized by a scale factor of 10,000 and log1p-transformed using *LogNormalize()* before identifying variable genes. Data were scaled, centered, and principal components were identified as detailed above. Data were then integrated using Harmony^20^ (v.0.1.0) followed by Nearest Neighbor analysis (*FindNeighbors*) and dimensional reduction using uniform manifold approximation and projection (*RunUMAP).* Cell clusters were identified using *FindClusters*.

#### 4) Visium spatial gene expression data processing and analysis using Cell2location

##### i. Cell-type deconvolution of spatial transcriptomics data using Cell2location^13^

To spatially resolve cell populations identified in the kidney, pre- and post-transplant, we used Cell2location^13^, a Bayesian model which estimates cell type abundance by deconvoluting a spatial expression count matrix into a set of reference cell type signatures. The model takes a spatial expression matrix with mRNA counts of genes at spatial locations and a matrix of reference cell type signatures as input. All Cell2location analyses were conducted using Scanpy^21^ (v. 1.9).

##### ii. Construction of snRNA-seq and scRNA-seq reference transcriptomes

Reference transcriptomes input into Cell2location derived from the sorted CD45+ cells (scRNA-seq) (n = 6,513) and the nuclei isolated from the four biopsies of the porcine kidney taken pre-transplant and on Day1, Day3 and Day 3T post-transplant (n = 7,868). These data were pre-processed as described above for scRNA-seq. Cells from both datasets were merged into a unified object using Seurat’s *merge* function yielding a total of 14,381 cells and 27,536 genes in the reference dataset. The merged dataset was then normalized, scaled, and centered as described above. Thirty principal components were found and used to construct a k-nearest neighbor (KNN) graph and clustering was performed using modularity optimization, resulting in 17 reference cell clusters which were ultimately collapsed to broader cell types (n=13 clusters). Cell clusters were visualized in low-dimensional space on a UMAP, and marker genes were used to annotate each cell cluster in the reference dataset (Extended Fig. 8).

##### iii. Estimating reference cell type signatures

*The snRNA-seq and scRNA-seq reference data* and corresponding reference cell type annotations were loaded into Scanpy using *sc.read_10x_mtx*. Gene selection and general QC were conducted as described before. Expression signatures of the 13 reference cell types in the reference dataset were then estimated using the negative binomial regression model while accounting for batch effects. The created model was further trained to estimate the reference cell type signatures on all cells in the dataset (train_size=1), mini-batch size = 2,500 and a training duration of 600 epochs, with GPU acceleration. Model accuracy was evaluated based on the evidence lower bound (ELBO) loss and reconstruction accuracy plots.

##### iv. Spatial mapping of kidney reference cell-types to spatially barcoded xenotransplant biopsies using Cell2location

We imported the Visium spatial transcriptomics data for each biopsy from 10X Space Ranger into Scanpy, using *scanpy.read_visium.* Mitochondrial genes were identified and removed. Before running Cell2location to map our reference cell types to each Visium dataset, both the estimated reference cell type signatures (gene expression), and spatial data from each respective biopsy were filtered to select genes shared between the two data objects. The filtered objects were both used as input to Cell2location.

We specified two hyperparameters required to run Cell2location: 1) The expected cell abundance per location (N_cells_per_location), which was estimated by manually counting nuclei in 10 random capture spots from the respective H&E images and averaging these values to give us the average cell abundance per tissue sample (Pre-transplant = 6.6 cells/capture spot; Day 1 = 5.7 cells/capture spot; Day 3 = 5.6 cells/capture spot; Day 3T = 5.4 cells/capture spot); and 2) a parameter which accounted for within-experiment variation in RNA detection sensitivity (*detection_alpha*). We set detection alpha = 20 in order to ensure the greatest accuracy and sensitivity given that strong gradients in mRNA detection sensitivity are commonly observed in adult human 10x Visium data.

After providing the reference cell type signatures, spatial data, and hyperparameters specified above as inputs, the Cell2location model was trained using max_epochs = 30000 on the full dataset (batch_size = None) while estimating cell abundance at all locations (train_size = 1). We plotted ELBO loss history during training and assessed mapping quality by examining reconstruction accuracy plots. We conducted four runs using the same reference dataset for each biopsy taken at the four different time points (Pre-transplant, Day1, Day3 and Day 3T). We exported estimated posterior distributions of cell abundance (‘num_samples’: 1000, ‘batch_size’: mod.adata.n_obs, ‘use_gpu’: True) and added a 5% quantile of the posterior distribution, representing the value of cell abundance corresponding to high confidence in the model. We further leveraged Scanpy’s plotting function *scanpy.pl.spatial* to visualize spatial scatter plots of cell type abundance in spatial coordinates.

#### 5) Spatially barcoded libraries prepared from control kidney samples, including human kidney, 10-GE pig kidney, and wild-type pig kidney.

##### i. Data pre-processing

Base call (BCL) files were converted to FASTQ files using the Space Ranger version 1.3 *mkfastq* function. Space Ranger *count* function was used to align the FASTQ files to the hybrid hg38-ss11 genome to generate count matrices.

##### ii. Data Analysis

Spatial transcriptomics analyses were carried out using packages created for the R statistical analysis environment (v. 4.2.1). Data were primarily analyzed using Seurat (v. 4.2.0) and its associated dependencies. Data from each sample were imported and structured into a Seurat object using the *Load10X_Spatial*. Data were further filtered (nCount_Spatial > 1) and normalized using *SCTransform* before visualizing gene expression in the respective spatial transcriptomics landscapes.

### Species assignment validation analyses using the modified species-specific reference genome

#### 1) Datasets

- scRNA-seq libraries prepared from pre-transplant porcine and human PBMCs
- scRNA-seq library prepared from CD45+ immune cells sorted from the explanted xenografts

#### 2) Construction of the species-specific reference genomes & data analysis

Sequenced reads were processed with Cell Ranger (version 6.1.1). Reference genomes utilized included the hg38 and ss11 genomes as provided by Ensembl as well as the pre-compiled hg38 reference genome provided by 10x Genomics. Pre-transplantation porcine and human samples were initially mapped to the opposite species’ reference genome to identify genes. Any gene with > 3 counts assigned was then subsequently identified and removed from the original reference, thereby resulting in a modified reference genome for each species. All samples were subsequently mapped to both species’ modified reference genomes and gene mapping rates on a per cell basis were compared between the two to enable identification of porcine cells from human. As some level of mapping consistently occurred when processing samples against the opposite species’ reference genome, cells were identified as porcine if they had a ratio of human to porcine mapped genes < 0.75 and subsequently identified as human if the ratio was > 1.33. Cells that fell between the two ratios were labeled as ambiguous and excluded from analysis. Samples were then mapped to the original unmodified reference genomes of each species. For post-transplantation human blood samples, cells identified as porcine from the modified reference mapping, were filtered out from the unmodified human reference mapped data and included from the unmodified porcine reference mapped data for final analysis. Downstream analysis was completed using R (v.4.2.0) and Seurat (v.4.1.1) using default parameters unless otherwise noted. Samples were normalized and scaled after standard QC measures including filtering of cells with less than 200 features, removing features with less than 3 cells expressing them, removing immunoglobulin genes and removing cells that had over 8% mitochondrial gene expression. Samples were integrated using Harmony (v.0.1).

### BLAST analysis

Nucleotide basic local alignment search tool algorithm blastn (https://blast.ncbi.nlm.nih.gov) was used to generate pairwise alignments of human and porcine sequences.

## Data Availability

The data from this study are available on request from the corresponding author. The data are not publicly available due to privacy restrictions. All analyses were performed using open source packages, and details of commands used in the packages are provided in the Methods.
